# Cardiac surgery dilemmas: evaluating outcomes of sternotomy versus thoracotomy

**DOI:** 10.55730/1300-0144.5992

**Published:** 2024-12-24

**Authors:** Muhammet Fethi SAĞLAM, Emrah UĞUZ, Kemal Eşref ERDOĞAN, Hüseyin Ünsal ERÇELİK, Murat YÜCEL, Mete HIDIROĞLU, Erol ŞENER

**Affiliations:** 1 Department of Cardiovascular Surgery, Faculty of Medicine, Ankara Yıldırım Beyazıt University, Ankara, Turkiye; 2 Department of Cardiovascular Surgery, Ankara Bilkent City Hospital, Ministry of Health, Ankara, Turkiye

**Keywords:** Sternotomy, thoracotomy, tricuspid valve replacement, cardiac surgery

## Abstract

**Background/aim:**

This retrospective study aimed to compare the clinical outcomes, including reoperation rates, mortality, and valve size measurements, in patients undergoing sternotomy and thoracotomy for cardiac surgery, particularly focusing on tricuspid valve replacements. The study sought to highlight differences in surgical approaches and their impact on patient outcomes.

**Materials and methods:**

A total of 107 patients were included, with 82 undergoing sternotomy and 25 undergoing thoracotomy. Preoperative right ventricular function parameters (mean pulmonary artery pressure, tissue Doppler imaging, and tricuspid annular plane systolic excursion), valve sizes, and other clinical data were recorded. Statistical analyses were performed to assess differences in outcomes between the two surgical groups.

**Results:**

Reoperation rates were significantly higher in the sternotomy group than in the thoracotomy group. Additionally, there were notable differences in valve sizes between the groups, with larger valves being used in sternotomy cases. However, mortality rates did not differ significantly between the groups.

**Conclusion:**

This study suggests that while sternotomy may lead to higher reoperation rates, the two surgical approaches yield comparable mortality outcomes. Further prospective studies are needed to fully understand the long-term implications of these findings.

## 1. Introduction

Cardiac surgery has undergone significant changes in recent years due to advances in technology and surgical techniques. However, debates continue among cardiac surgeons as to which technique is superior, especially among major surgical approaches such as sternotomy and thoracotomy [1–3]. While sternotomy is considered the gold standard for open heart surgery because it provides full access to the heart, thoracotomy is a less invasive option and is preferred, especially in minimally invasive surgical procedures. Both methods have advantages and disadvantages and there is no clear consensus on which approach is more suitable for patients [4–6].

In the literature, studies comparing sternotomy and thoracotomy methods have generally focused on mitral and aortic valve operations [1,7]. However, outcomes specific to tricuspid valve replacement have been addressed in fewer studies; therefore, the available information is limited. Most studies suggest that the differences between sternotomy and thoracotomy are not significant or that complication rates are higher after sternotomy, whereas results after thoracotomy may be preferable because it is less invasive. Especially in this group of patients, the choice of surgical approach may have a decisive influence on their postoperative outcome [8].

In the present study, we analyzed in detail the differences in reoperation and mortality rates and other clinical parameters in patients who underwent tricuspid valve replacement via sternotomy and thoracotomy. Our study compared not only reoperation and mortality rates but also preoperative right ventricular function, valve measurements, and other important clinical data. Our aim was to make an important contribution to the literature by determining which surgical technique is more advantageous in patients undergoing tricuspid valve replacement and to help cardiac surgeons make more informed decisions in the choice of surgical approach.

In this context, the most important difference between our study and the literature is that it provides a comprehensive comparison specific to tricuspid valve replacement and is analyzed over a large dataset. These results provide important findings that may help in making more informed decisions regarding the choice of surgical technique.

## 2. Materials and methods

### 2.1. Study design and patient selection

This retrospective cohort study was approved by the 2nd Clinical Research Ethics Committee of Ankara Bilkent City Hospital on 07.06.2023, and the ethics committee decision number was recorded as E2-23-4294. The study was conducted with data from patients who underwent tricuspid valve replacement between 2019 and 2023. The aim of the study was to compare the effects of sternotomy and thoracotomy surgical approaches on preoperative right ventricular function, the need for reoperation, mortality rates, and other clinical parameters. Patient data were obtained retrospectively from patient records and selected according to appropriate criteria.

### 2.2. Patient criteria

The inclusion criteria were being 18 years of age or older, having undergone tricuspid valve replacement surgery, and having complete clinical and surgical records. Patients who had undergone additional heart surgery, who could not be evaluated before surgery due to serious comorbidities, or who had incomplete records were excluded.

A total of 107 patients (82 who underwent sternotomy, 25 who underwent thoracotomy) who met these criteria were included in the study.

### 2.3. Surgical methods and clinical parameters

The patients in the study were divided into two main groups: those who underwent sternotomy and those who underwent thoracotomy. The sternotomy group included surgical interventions performed by opening the thorax through an incision made at the median sternal line. The thoracotomy group included standard or minimally invasive thoracotomy techniques performed through an incision in the right or left intercostal space. In both groups, surgical procedures were performed by the relevant specialist surgeons and standard preoperative and postoperative protocols were applied.

### 2.4. Data collection and clinical parameters

The clinical parameters analyzed in the study included preoperative right ventricular function, preoperative mean pulmonary artery pressure (MPAP), preoperative tissue Doppler imaging (TDI), preoperative tricuspid annular plane systolic excursion (TAPSE), valve measurements (valve diameter and valve type), the need for reoperation, mortality rates, and other clinical parameters, pace requirement, and exploration tricuspid regurgitation (TR) classification. These parameters provide detailed information about the surgical process and its outcome, allowing comparison of the efficacy and outcomes of both surgical approaches.

### 2.5. Statistical analysis

SPSS 29.0 was used to evaluate the data. Categorical data are shown with frequency and percentage values, whereas numerical data are shown with mean and standard deviation values. The chi-squared test was used for categorical data analysis, and the Mann-Whitney U test, which is a nonparametric test, was used to determine the difference between numerical data and the type of surgical method since the number of patients in whom thoracotomy was used was less than 30. Fisher’s exact value was used in the chi-squared test when there were fewer than 5 observations. In cases where a significant relationship was found as a result of the chi-squared test, post hoc analysis with Bonferroni correction was performed, and the results are indicated with the letters a and b. The statistical significance level was p < 0.05 for all tests.

## 3. Results

As shown in Table 1, there was no statistically significant difference in sex, preoperative right ventricular function, valve type, type of valve, mortality, or pace marker requirement between sternotomy and thoracotomy, which are two methods used in cardiac surgery. The mean age of the patients who underwent sternotomy was 54.28 ± 11.89 years, whereas the mean age of those who underwent thoracotomy was 53.08 ± 11.78 years. According to the Mann-Whitney U test, no significant difference was found between age and surgical method. No statistically significant relationship was found between sex and the surgical method used (p > 0.05). The proportions of women who underwent sternotomy (72.0%) and who underwent thoracotomy (76.0%) were very close to each other. Similarly, no significant differences were found in preoperative right ventricular function, valve type, TR, mortality, or space requirement between sternotomy and thoracotomy (p > 0.05). A significant difference was found only in expansion between sternotomy and thoracotomy (p < 0.05). While 63.4% of the 82 patients in the sternotomy group had resternotomy, in the thoracotomy group 28% of the 25 patients had minimal thoracotomy.

In accordance with Table 2, the Mann-Whitney U test was used to analyze whether there was a statistically significant difference in preoperative MPAP, preoperative TDI, preoperative TAPSE, and valve measurements between sternotomy and thoracotomy. The mean preoperative MPAP of the sternotomy patients was 31.94 ± 10.02 mmHg, whereas that of the thoracotomy patients was 31.76 ± 8.68 mmHg. The preoperative MPAP values of the patients who underwent surgery with the two different methods were not significantly different (p > 0.05). Similarly, the difference between the preoperative TDI and preoperative TAPSE values was not significant (p > 0.05). While mean valve size was larger in the patients who underwent sternotomy, it was lower in those who underwent thoracotomy and this difference was significant (p < 0.05). The mean valve size in the sternotomy patients was 31.18 ± 1.70 mm, whereas in the thoracotomy patients it was 29.96 ± 1.65 mm. The valve measurements of the thoracotomy patients were significantly lower than those of the sternotomy patients.

A comparison of the reoperation and mortality rates is shown in Figure.

## 4. Discussion

In the present study, demographic and clinical characteristics and preoperative right ventricular function and valve measurements were compared between patients who underwent cardiac surgery via sternotomy and those who underwent thoracotomy. The results revealed significant differences between the two surgical methods, especially in terms of the rates of exploration and valve measurements. It is important to examine in depth how these findings agree with or differ from those of other studies in the literature.

The effects of sternotomy and thoracotomy on clinical outcomes have been extensively studied in the literature [9,10]. Although sternotomy is preferred, especially because it provides complete cardiac access, it is often emphasized in the literature that reoperation and complication rates may be higher [11,12]. For example, Pineda et al. reported higher reoperation rates in patients who underwent sternotomy than in those who underwent thoracotomy [12]. In the present study, higher reoperation rates were observed in the sternotomy group, which is consistent with the literature. The greater risk of complications after sternotomy may also be due to the more invasive nature of this method [13].

The effects of thoracotomy on valve measurements may be considered a reflection of its minimally invasive nature. The fact that valve measurements were significantly lower in the patients who underwent thoracotomy suggests that minimally invasive surgical techniques may cause less tissue damage. This finding is consistent with the results of the study by Khalid et al. [14], who reported that minimally invasive surgery accelerated the postoperative recovery process and reduced the risk of complications. Therefore, the less invasive approach of thoracotomy may have a favorable impact on surgical outcomes.

In terms of preoperative right ventricular function, no significant difference was found between the two groups in our study, which is consistent with the literature in this field. In the study by Singh et al. [15], no significant difference was found between the sternotomy and thoracotomy methods in terms of right ventricular function. However, some studies have reported that the negative effects of the sternotomy method on the right ventricle are more pronounced [16]. These conflicting findings may be due to differences in surgical techniques, the diversity of patient populations, or the retrospective nature of the study. Therefore, prospective studies are needed to more clearly understand the effects of surgical methods on right ventricular function.

The analysis of a large group of patients in our study increases the generalizability of our findings to a wider population. This is one of the strengths of our study. In addition, the large number of subjects increases the statistical power of the data obtained and reinforces the reliability of the results. However, the limitations of the retrospective design and the comparison of only two surgical methods partially limit the generalizability of our study. Future prospective and multicenter studies will allow more robust confirmation of these findings and a more comprehensive evaluation of different surgical techniques.

In conclusion, the effects of sternotomy and thoracotomy on clinical outcomes were evaluated in a large group of patients in our study, and the results were generally consistent with those of other studies in the literature [17,18]. Further research is needed to optimize the choice of surgical methods and postoperative management strategies. Our study may provide an important reference point for future studies in this field and may help cardiac surgeons make more informed decisions in their choice of surgical approach.

## Figures and Tables

**Figure:**
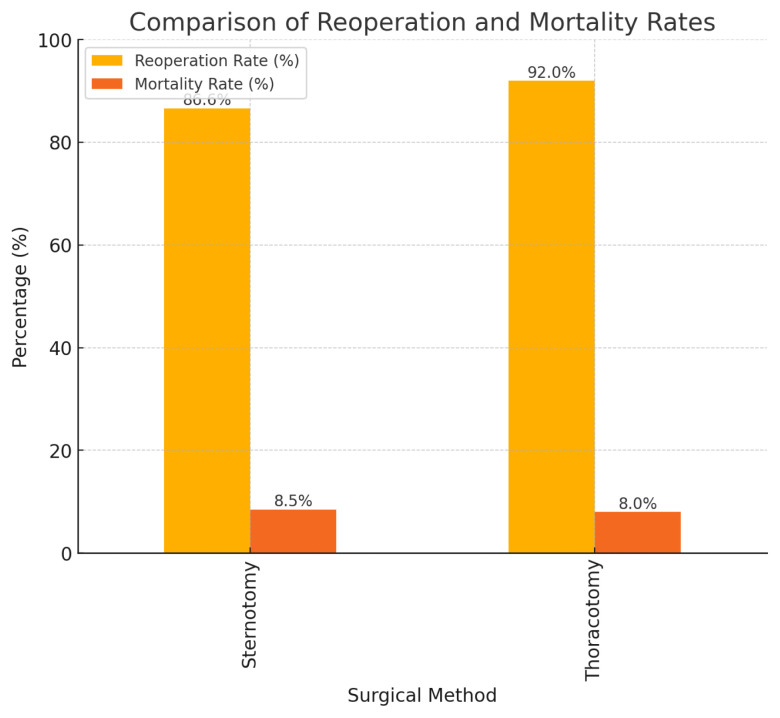
Comparison of reoperation and mortality rates. This figure illustrates the comparison of reoperation and mortality rates between patients undergoing sternotomy and thoracotomy. The reoperation rates were higher in both groups, with sternotomy having a reoperation rate of 86.6% and thoracotomy one of 92.0%. The mortality rates were relatively low and similar between the groups, with sternotomy showing a mortality rate of 8.5% and thoracotomy one of 8.0%. These findings suggest that while these surgical methods have comparable mortality rates, thoracotomy may be associated with slightly higher reoperation rates compared to sternotomy.

**Table 1:** Comparison of demographic and clinical characteristics of patients who underwent sternotomy and thoracotomy.

		Sternotomy		Thoracotomy			
		n	%	n	%	*χ* *^2^*	p
Age (*χ* ±SD). Median (min–max)		54.28±11.89	57 (18–76)	53.08±11.78	57 (20–68)	U=0.571	0.568
Sex	Male	23	28.0%	6	24.0%	0.159	0.690
	Female	59	72.0%	19	76.0%		
Reoperation	No	11	13.4%	2	8.0%	0.526	0.468
	Yes	71	86.6%	23	92.0%		
Exploration	Resternotomy	52^a^	63.4%	0^b^	0.0%	107.000	**<0.001**
	Sternotomy	30 ^a^	36.6%	0 ^b^	0.0%		
	Thoracotomy	0 ^a^	0.0%	18^b^	72.0%		
	Thoracotomy (minimal)	0 ^a^	0.0%	7 ^b^	28.0%		
Preoperative right ventricular function	Normal	18	22.0%	3	12.0%	1.471	0.689
	Mild depression	9	11.0%	4	16.0%		
	Middle depression	38	46.3%	12	48.0%		
	Serious depression	17	20.7%	6	24.0%		
Valve type	Bioprosthesis	34	41.5%	5	20.0%	3.810	0.051
	Metallic	48	58.5%	20	80.0%		
Tricuspid regurgitation	1TR	0	0.0%	1	4.0%	4.117	0.249
	2TR	6	7.3%	2	8.0%		
	3TR	17	20.7%	3	12.0%		
	4TR	59	72.0%	19	76.0%		
Mortality	Ex	7	8.5%	2	8.0%	0.007	0.933
	Alive	75	91.5%	23	92.0%		
The need for a pace marker	Yes	10	12.2%	5	20.0%	0.968	0.325
	No	72	87.8%	20	80.0%		

U = Mann-Whitney U test, χ^2^ = Chi-squared test, p < 0.05, TR = Tricuspid regurgitation.

* Fisher’s exact value was used when there were fewer than 5 observations.

The letters a and b indicate the results of post hoc analysis. The difference between different letters is significant.

**Table 2:** Comparison of preoperative right ventricular functions and valve measurements in patients undergoing sternotomy and thoracotomy.

	Sternotomy		Thoracotomy			
	*χ̄* ±SD	Median (min–max)	*χ̄* ±SD	Median (min–max)	U	p
Preoperative MPAP (mmHg)	31.94±10.02	30 (14–55)	31.76±8.68	30 (16–55)	0.011	0.991
Preoperative TDI	10.77±2.69	10.5 (6–18)	10.56±2.13	11.4 (7–16)	0.618	0.537
Preoperative TAPSE	16.60±3.30	16 (10–29)	16.56±2.35	16 (13–23)	0.551	0.582
Valve size (mm)	31.18±1.70	31 (27–33)	29.96±1.65	31 (27–33)	3.197	**<0.001**

U = Mann-Whitney U test, p < 0.05, Preoperative mean pulmonary artery pressure (MPAP), Tissue Doppler imaging (TDI), Tricuspid annular plane systolic excursion (TAPSE).

## Data Availability

The datasets used and/or analyzed during the current study are available from the corresponding author on reasonable request.
